# Epidermal Growth Factor Receptor (EGFR) gene copy number (GCN) correlates with clinical activity of irinotecan-cetuximab in K-RAS wild-type colorectal cancer: a fluorescence in situ (FISH) and chromogenic in situ hybridization (CISH) analysis

**DOI:** 10.1186/1471-2407-9-303

**Published:** 2009-08-27

**Authors:** Mario Scartozzi, Italo Bearzi, Alessandra Mandolesi, Chiara Pierantoni, Fotios Loupakis, Alberto Zaniboni, Francesca Negri, Antonello Quadri, Fausto Zorzi, Eva Galizia, Rossana Berardi, Tommasina Biscotti, Roberto Labianca, Gianluca Masi, Alfredo Falcone, Stefano Cascinu

**Affiliations:** 1 Clinica di Oncologia Medica, AO Ospedali Riuniti-Università Politecnica delle Marche, Ancona, Italy; 2 Anatomia Patologica, AO Ospedali Riuniti-Università Politecnica delle Marche, Ancona, Italy; 3 Oncologia Medica, Ospedale Civico, Livorno – Università degli Studi di Pisa, Italy; 4 Oncologia Medica, Fondazione Poliambulanza, Brescia, Italy; 5 Oncologia Medica, AO Parma, Italy; 6 Oncologia Medica, Ospedali Riuniti, Bergamo, Italy; 7 Anatomia Patologica Fondazione Poliambulanza, Brescia, Italy

## Abstract

**Background:**

K-RAS wild type colorectal tumors show an improved response rate to anti-EGFR monoclonal antibodies. Nevertheless 70% to 40% of these patients still does not seem to benefit from this therapeutic approach. FISH EGFR GCN has been previously demonstrated to correlate with clinical outcome of colorectal cancer treated with anti-EGFR monoclonal antibodies. CISH also seemed able to provide accurate EGFR GCN information with the advantage of a simpler and reproducible technique involving immunohistochemistry and light microscopy. Based on these findings we investigated the correlation between both FISH and CISH EGFR GCN and clinical outcome in K-RAS wild-type colorectal cancer treated with irinotecan-cetuximab.

**Methods:**

Patients with advanced K-RAS wild-type, colorectal cancer receiving irinotecan-cetuximab after failure of irinotecan-based chemotherapy were eligible.

A cut-off value for EGFR GCN of 2.6 and 2.12 for FISH and CISH respectively was derived from ROC curve analysis.

**Results:**

Forty-four patients were available for analysis. We observed a partial remission in 9 (60%) and 2 (9%) cases with a FISH EGFR GCN ≥ 2.6 and < 2.6 respectively (p = 0.002) and in 10 (36%) and 1 (6%) cases with a CISH EGFR GCN ≥ 2.12 and < 2.12 respectively (p = 0.03). Median TTP was 7.7 and 6.4 months in patients showing increased FISH and CISH EGFR GCN whereas it was 2.9 and 3.1 months in those with low FISH and CISH EGFR GCN (p = 0.04 and 0.02 respectively).

**Conclusion:**

FISH and CISH EGFR GCN may both represent effective tools for a further patients selection in K-RAS wild-type colorectal cancer treated with cetuximab.

## Background

The expanding role of anti-EGFR therapeutic modalities in colorectal cancer along with the growing number of cases potentially requiring such a treatment approach made the need for a correct and reliable identification of responding tumors even more relevant [1-7].

Since its early introduction, the use of K-RAS mutational status as a predictive factor for anti-EGFR targeted monoclonal antibodies seemed to possess the necessary potential for a full translation into clinical practice of the true concept of targeted therapy in this setting. Research data from retrospective series have in fact repeatedly demonstrated that a K-RAS mutant status was strictly correlated to clinical resistance to EGFR-targeted monoclonal antibodies [7-12]. Consequently the evaluation of K-RAS in colorectal tumors promised to allow a more accurate treatment selection with a consistent and highly desired reduction of unnecessary toxic effects and economic costs.

However if on the one hand we are now able to exclude from anti-EGFR treatment with monoclonal antibodies those patients with putative refractory colorectal tumors (i.e. those harboring a K-RAS mutation), on the other hand we are still incapable to accurately select responding patients among those without K-RAS mutations. Published data clearly suggest that a non negligible proportion of patients, ranging from 70% to 40% in different series, does not seem to benefit from the use of anti-EGFR targeted antibodies although in the absence of a mutation of the K-RAS gene (i.e. K-RAS wild-type) [7-12].

Along with other biological determinants (such as B-RAF mutation analysis) [12] EGFR gene copy number (GCN) as determined by fluorescence in situ hybridization (FISH) analysis seemed to correlate with response to anti-EGFR monoclonal antibodies in an initial study by Moroni et al [13]. These findings were subsequently confirmed in a further retrospective analysis from the same group of investigators but in a larger and more homogeneous series [14]. A more recent analysis investigating colorectal cancer patients receiving cetuximab showed once again that cases with high EGFR GCN have an increased likelihood to respond to therapy [15]. Taken together data from all these studies suggest a strong role for EGFR GCN in predicting the activity of EGFR targeted monoclonal antibodies in colorectal cancer patients. Nevertheless FISH technique is expensive, time consuming, and requires a special protocol, materials and fluorescent microscopy. In contrast chromogenic in situ hybridization (CISH), which utilizes a peroxidase reaction to detect the locus of interest, can be performed in the clinical immunohistochemistry laboratory and interpreted by standard light microscopy [16]. The reliability of CISH in detecting HER-2 gene amplification has been previously proved in breast tumors [17,18]. Furthermore this analysis technique seemed more recently able to provide EGFR GCN information as accurate as that supplied by FISH in non small cell lung cancer [19]. This technique for determination of EGFR GCN has been validated in colorectal tumors as well [20]. We then decided to investigate the correlation between both FISH and CISH EGFR GCN and clinical outcome in terms of response rate and time to progression (TTP) in irinotecan-refractory K-RAS wild-type colorectal cancer patients treated with irinotecan-cetuximab. This was done in order to identify a possible simplified molecular determinant of response in this group of patients.

## Methods

### Patients selection

Patients with histologically proven EGFR-positive, K-RAS wild-type, metastatic, colorectal cancer previously treated with an irinotecan-based chemotherapy regimen and receiving a combination of cetuximab and irinotecan were eligible for our retrospective analysis. To be eligible patients must also have received the irinotecan-based chemotherapy regimen for at least 6 weeks and must have presented progression of disease during receipt of this regimen or within three months thereafter. All patients received cetuximab at an initial dose of 400 mg per square meter followed by weekly infusions of 250 mg per square meter. Irinotecan was administered at a dose of 180 mg per square meter every 2 weeks either alone or in combination with 5 fluorouracil and leucovorin. Tumor response was evaluated every 8 weeks by clinicians' assessment and according to the Response Evaluation Criteria in Solid Tumors (RECIST). This study was approved by the institutional ethics committee. Informed consent was obtained from participants of the study.

### K-RAS mutational analysis

Formalin-fixed and paraffin-included tumor samples were analyzed for KRAS exon 2 mutations, located within the codon 12 and 13 (Gly12Asp, Gly12Ala, Gly12Val, Gly12Ser, Gly12Arg, Gly12Cys and Gly13 Asp).

For each tumor sample the neoplastic area was selected and DNA was extracted using the DNA extraction kit from QIAGEN (QIAamp^® ^DNA Mini and Blood Mini). The DNA extracted was PCR amplified using the following sense and antisense primer: 5'-AAGGCCTGCTGAAAATGACTG-3' and 5'-CAAAGAATGGTCCTGCACCAG-3'.

After the purification using QIAquick^® ^PCR Purification kit, the PCR products were direct sequenced with Big Dye V1.1 Terminator Kit (Applied Biosystems, Foster City, CA, USA) and an ABI Prism 3100 DNA sequencer (Applied Biosystems).

### FISH analysis

Analysis of EGFR amplification was performed using the standard dual-colour EGFR Spectrum Orange™/CEP7^® ^Spectrum Green™ probe (Visys, Downers Grove, IL USA). In brief, paraffin sections were deparaffinizzed, dehydrated in ethanol and air-dried. After treatment in 0,05% pepsin/0,1 N HCL for 45 min at 37°C, the sample were aged in 0,1% NP-40/2 × SSC (Standard Saline Citrate solution) for 10 min at 37°C and DNA was denatured by treatment in 70% formamide/2 × SSC for 4 min at 85°C. A measure of 5 μl of the probe solution was then placed on a glass slide with a coverslip. The sample slides were hybridized overnight at 37°C and washed in 0,4 × SSC/0,3% NP-40 at 73°C for 2 min. Nuclei were counterstained with 4',6-diamino-2-phenylindole dihydrochloride and p-phenylenediamine in phosphate-buffered saline and glycerol (DAPI II) (Vysis). Each FISH assay included normal breast tissue sections, as negative control, and sections of breast cancer previously confirmed to have amplification of EGFR as positive control.

FISH analysis was performed using a fluorescence microscope (Nikon Optphot-2 and Quips Genetic Workstation) equipped with the Tripple Bandpass Filter set (Vysis) for DAPI II, SpectrumOrange and SpectrumGreen. Filter was specifically set to SpectrumOrange and SpectrumGreen.

Only individual and well delineated cells were scored; overlapping cells were excluded from the analysis. At least 60 cells were scored for each case or control sample.

Each tumor was assessed by the average (GCN), the maximum numbers of the copies of EGFR gene per cell, the average EGFR/chromosome 7 copy number (CEP7) ratio and ploidy.

Amplification was defined as ratio of EGFR signals to chromosome 7 centromere signals of 2 or more. Polyploidy was defined as a mean of >2 chromosome 7 signals, per cell. The percentage of the cells showing polyploidy and EGFR amplification (% CEP7 >2; % EGFR/CEP 7 ≥ 2) was estimated for each tumor.

FISH results were evaluated independently by two pathologist (IB and AM) who were blinded about clinical outcome.

### CISH analysis

Chromogenic in situ hybridization for the EGFR gene was performed according to manufacturer's instructions (Zymed Laboratories Inc., South San Francisco, California, USA).

Briefly the sections of the formalin-fixed and paraffin-embedded tissue were incubated at 55°C overnight. The slides were then deparaffinizzed in xylene and graded ethanols. Pre-treatment heating was carried out in the pre-treatment buffer (Zymed Laboratories Inc.) at 96°C for 15 min.

The tissue was digested with pepsin for 10 min at room temperature, subsequently it was washed with deionised water, dehydrated with graded ethanol and air dried.

After application of Zymed Spot-Light^® ^oligoxigenin labeled EGFR probe (Zymed Laboratories Inc.), the slides were coverslipped and edges sealed with rubber cement. The slides were heated at 92°C for 5 min followed by overnight incubation at 37°C using moisturized chamber.

Post hybridization wash was performed the next day and followed by immunodetection using the CISH™ polymerdetection Kit (Zymed Laboratories Inc.).

The CISH signals were seen as dark brown dots and counted in at last 100 nuclei with a light microscopy using a × 40 objective. Only individual and well delineated cells were scored; while overlapping cells were excluded from the analysis. Also the average EGFR gene copies per nucleus for each tissue sections was calculated (EGFR GCN).

According to previous reports [16-20], a gene copy number of six o more in the nucleus in more than 50% of tumor cells was taken to indicate amplification of the EGFR gene. Cases with three to five gene copies were considered aneuploid.

CISH results were evaluated independently by two pathologist (IB and AM) who were blinded about clinical outcome.

### Statistical Analysis

Receiver operating characteristics (ROC) curve analysis was performed to determine a cut off value for FISH and CISH EGFR GCN continuous variables.

The association between EGFR FISH and CISH GCN and response rate was estimated by Fisher's exact test.

Survival distribution was estimated by the Kaplan-Meier method. Significant differences in probability of relapsing between the strata were evaluated by log-rank test.

For statistical analysis overall survival (OS) and time to progression (TTP) were defined respectively as the interval between the start of cetuximab and irinotecan therapy to death or last follow-up visit and as the interval between the start of cetuximab and irinotecan therapy to clinical progression or death or last follow up visit if not progressed.

Statistical significance was set at p < 0.05 for each analysis.

## Results

Forty-four patients were eligible for our analysis, 23 males (52%%) and 21 females (48%), median age at diagnosis was 66 (range 39–78). Fourteen patients (32%) received cetuximab-irinotecan therapy as a second-line treatment, the remaining 30 cases (68%) were treated with cetuximab-irinotecan after failure of at least 2 previous lines of chemotherapy. Overall we observed a partial response in 11 patients (25%) and progressive disease in 21 cases (48%). Twelve patients (27%) showed a tumor stabilization, while no complete remissions were obtained. Median time to progression for the whole patients population was 3.6 months and median overall survival was 10.6 months (Table 1). All patients but 3 (9%) received cetuximab in combination with irinotecan alone.

**Table 1:** Patients characteristics and global results for FISH/CISH EGFR GCN analysis and clinical outcome.

	**Total Population**	**EGFR FISH GCN** ≥ **2.6**	**EGFR FISH GCN** **< 2.6**	**P**	**EGFR CISH GCN** = **2.12**	**EGFR CISH GCN** **< 2.12**	**p**
	
**Number (%)**	44	15 (40)	22 (60)		28 (64)	16 (36)	
**Sex (%)**							
	
Male	23 (52)	10 (67)	13 (60)		15 (53)	9 (56)	
Female	21 (48)	5 (33)	9 (40)		13 (47)	7 (44)	
**Median age (range)**							
	
	66 (39–78)	66 (39–78)	66 (39–78)		65 (39–78)	66 (40–78)	
**Previous lines of Chemotherapy (%)**							
	
1	14 (32)	5 (33)	8 (36)		8 (29)	6 (37)	
> 2	30 (68)	10 (67)	14 (64)		20 (71)	10 (63)	
**Response Rate (%)**							
	
Complete Remission	0 (0)	0 (0)	0 (0)		0 (0)	0 (0)	
Partial Remission	11 (25)	9 (60)	2 (9)	0.002	10 (36)	1 (6)	0.03
Stable Disease	12 (27)	5 (33)	8 (32)		10 (36)	2 (13)	
Progressive Disease	21 (48)	1 (7)	13 (59)	0.001	8 (30)	13 (81)	0.01
Not assessable	0 (0)	0 (0)	0 (0)		0 (0)	0 (0)	
**Survival (Months)**							
	
Time to Progression	3.6	7.7	2.9	0.04	6.4	3.1	0.02
Overall Survival	10.6	16	9.5		10.6	10.3	

Only statistically significant p values have been included.

**Figure 1:**
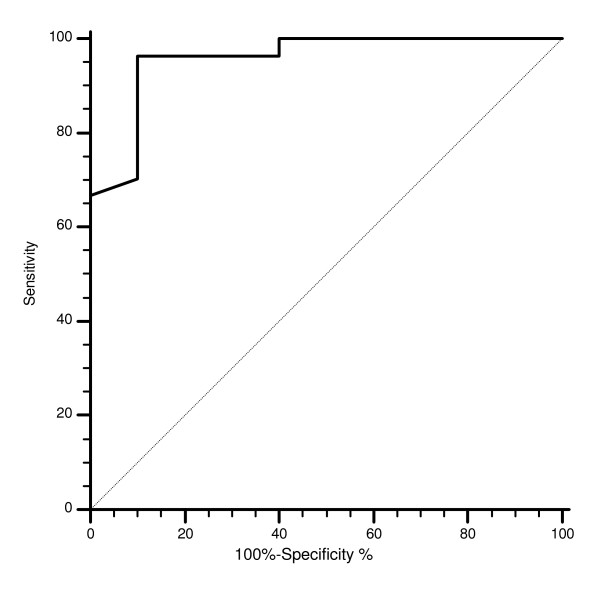
**Receiver operating characteristics (ROC) analysis based on mean FISH EGFR gene copy number with response to cetuximab therapy as end point**. In this model sensitivity was 96.3% (95% CI 81 – 99.4) and specificity was 90% (95% CI 55.5–98.3). AUC was 0.95, p = 0.0003.

FISH and CISH analysis for EGFR GCN was performed in primary tumors in all cases. In our series we observed only one case of EGFR amplification as defined by FISH (3%) and 1 case as defined by CISH (2%). FISH EGFR polyploidy was detected in 18 cases (48%). No correlation between FISH EGFR polyploidy, response rate, time to progression and overall survival was evident. The cut-off point with the highest sensitivity and specificity for estimating FISH EGFR GCN was set at 2.6 after ROC curve analysis (Figure 1).

FISH analysis was possible in 37 cases.

FISH EGFR GCN ≥ 2.6 was present in 15 (40%) colorectal tumors, whereas it was < 2.6 in the remaining 22 (60%) patients (table 1).

**Figure 2:**
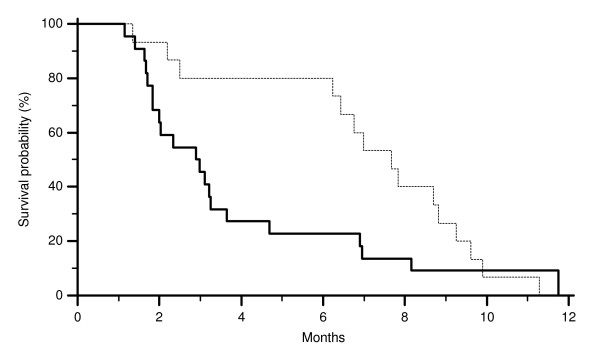
**Time to progression (TTP) of colorectal cancer patients showing FISH EGFR GCN ≥ 2.6 (-------) and FISH EGFR GCN < 2.6 (———) (p = 0.04)**.

**Figure 3:**
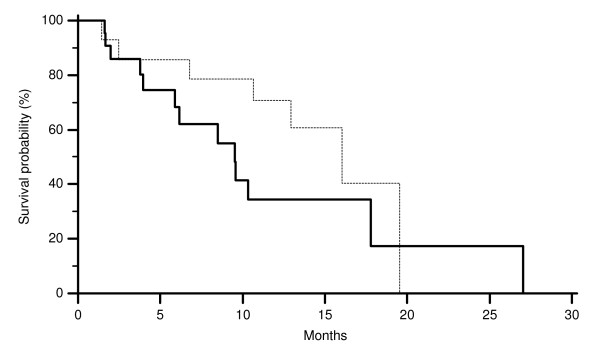
**Overall survival (OS) of colorectal cancer patients showing FISH EGFR GCN ≥ 2.6 (-------) and FISH EGFR GCN < 2.6 (———) (p = 0.2)**.

All major clinical characteristics resulted comparable among the two groups of patients (FISH EGFR GCN ≥ 2.6 and < 2.6). In particular no differences were noticed for sex, age at diagnosis and previous lines of chemotherapy (Table 1). On the contrary in patients showing a FISH EGFR GCN ≥ 2.6 we observed 9 partial remissions (60%) whereas 2 partial remission (9%) was observed in those showing a FISH EGFR GCN < 2.6 (p = 0.02). Accordingly FISH EGFR GCN ≥ 2.6 was also associated with a lower progression rate compared to FISH EGFR GCN < 2.6. In fact progressive disease was observed in 1 (7%) and 13 (59%) cases respectively (p = 0.001) (Table 1). Median time to progression was 7.7 months in patients showing FISH EGFR GCN ≥ 2.6 and 2.9 months in those showing FISH EGFR GCN < 2.6 (p = 0.04) (Figure 2). No statistically significant difference was noticed in median overall survival (Figure 3).

CISH analysis was possible in 44 cases.

**Figure 4:**
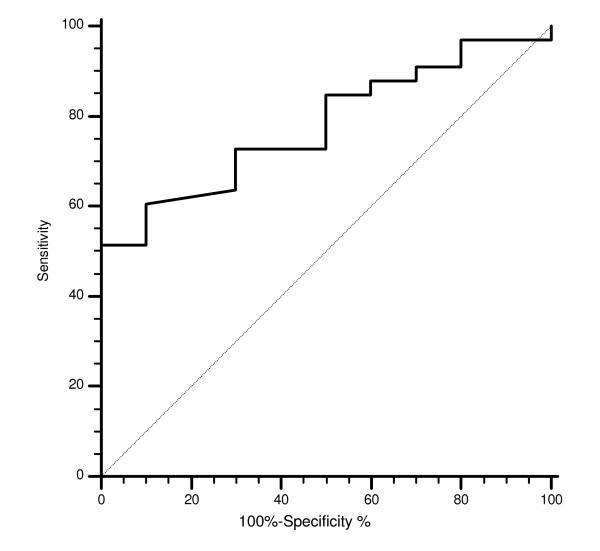
**Receiver operating characteristics (ROC) analysis based on mean CISH EGFR gene copy number with response to cetuximab therapy as end point**. In this model sensitivity was 51.5% (95% CI 33.6–69.2) and specificity was 100% (95% CI 69–100). AUC was 0.77, p = 0.002.

The cut-off point with the highest sensitivity and specificity for estimating CISH EGFR GCN was set at 2.12 after ROC curve analysis (Figure 4).

EGFR GCN ≥ 2.12 was present in 28 (64%) colorectal tumors, whereas it was < 2.12 in the remaining 16 (36%) patients.

**Figure 5:**
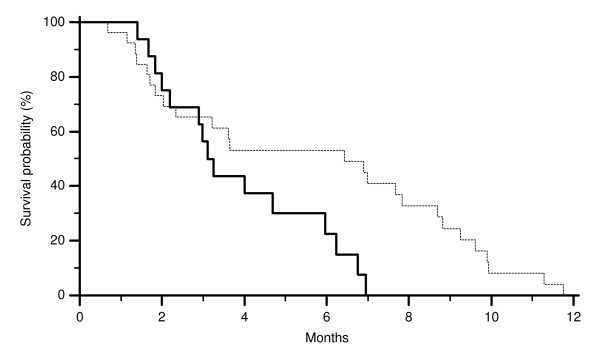
**Time to progression (TTP) of colorectal cancer patients showing CISH EGFR GCN ≥ 2.12 (-------) and CISH EGFR GCN < 2.12 (———) (p = 0.02)**.

**Figure 6:**
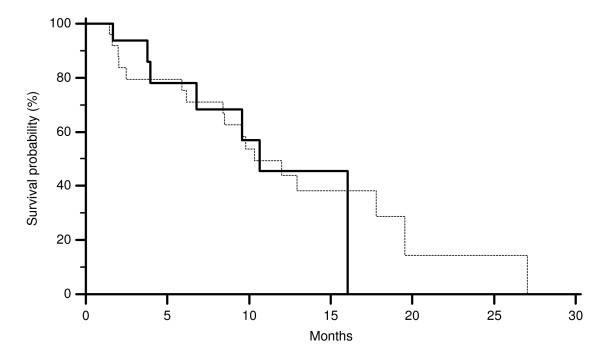
**Overall survival (OS) of colorectal cancer patients showing CISH EGFR GCN ≥ 2.12 (-------) and CISH EGFR GCN < 2.12 (———) (p = 0.95)**.

All major clinical characteristics resulted comparable among the two groups of patients (EGFR GCN ≥ 2.12 and < 2.12). In particular no differences were noticed for sex, age at diagnosis and previous lines of chemotherapy (Table 1). On the contrary in patients showing a CISH EGFR GCN ≥ 2.12 we observed 10 partial remissions (36%) whereas 1 partial remission (6%) was observed in those showing a CISH EGFR GCN < 2.12 (p = 0.03). Accordingly CISH EGFR GCN ≥ 2.12 was also associated with a lower progression rate compared to CISH EGFR GCN < 2.12. In fact progressive disease was observed in 8 (30%) and 13 (72%) cases respectively (p = 0.01) (Table 1). Median time to progression was 6.4 months in patients showing CISH EGFR GCN ≥ 2.12 and 3.1 in those showing CISH EGFR GCN < 2.12 (p = 0.02) (Figure 5). No statistically significant difference was noticed in median overall survival (Figure 6). In all responding patients evaluation of both EGFR FISH and CISH GCN was possible. An evident correspondence between results was observed in all but one case (Table 2).

**Table 2:** Correlation results for EGFR FISH and CISH GCN in patients responding to treatment

Case Number	EGFR FISH GCN ≥ 2.6	EGFR CISH GCN ≥ 2.12
3	2.6	2.12
10	2.9	2.13
13	1.48	2.09
19	2.6	2.78
20	3.03	4.03
22	2.7	3.18
26	2.6	2.43
29	2.83	5.15
31	3.85	2.96
35	1.92	2.63
37	2.9	2.6

In all responding patients evaluation of both EGFR FISH and CISH GCN was possible and an evident correspondence between results is observable in all but one case.

## Discussion

Biologically targeted agents for the treatment of colorectal cancer held along with an improved therapeutic level, the promise for a more accurate selection of patients candidate for such a treatment. The use of the anti-EGFR monoclonal antibodies in the clinical practice seemed to confirm the activity profile previously suggested [1-3]. Unfortunately the introduction of anti-EGFR monoclonal antibodies made also rapidly clear to the clinicians that a reliable predictive factor for outcome was, in fact, lacking [3-7]. The introduction of K-RAS mutational status analysis allowed a reliable selection of resistant patients (i.e. those with mutated K-RAS). However not all K-RAS wild-type cases were also responders to anti-EGFR monoclonal antibodies. This observation made the need for further selection even more crucial [7-12]. EGFR gene copy number as determined by FISH analysis seemed to correlate with response to anti-EGFR monoclonal antibodies in previous studies [13-15]. Taken together data from all these studies suggest a strong role for FISH EGFR GCN in predicting the activity of EGFR targeted monoclonal antibodies. Furthermore a previous report also suggested that EGFR GCN analysis may help identifying responding patients among wild-type colorectal cancer patients [15]. In the present analysis FISH EGFR GCN ≥ 2.6 correlated with improved response rate and time to progression, confirming its prominent role in predicting clinical outcome to cetuximab therapy even among K-RAS wild-type colorectal tumors and globally suggesting the possibility to further identify responding patients with the use of combined K-RAS mutation and FISH EGFR GCN analyses. Chromogenic in situ hybridization (CISH), may represent a further improvement in this setting. Unlike breast tumors, colorectal cancer cells often show an overlapping growth pattern in the examination specimen making FISH analysis less reproducible and harder to interpret. The CISH technique seems able to overcame this potentially confounding factor as it offers the opportunity to morphologically identify overlapping tumor cells, which can be consequently excluded from the analysis. In our series CISH EGFR GCN was also able to identify colorectal cancer patients more likely to respond to irinotecan-cetuximab therapy. In fact patients showing CISH EGFR GCN ≥ 2.12 obtained an interesting 37% response rate whereas response rate was 0% in those with a CISH EGFR GCN < 2.12 (p = 0.0007). Progression rate was also influenced by CISH EGFR GCN (30% vs. 72% in EGFR GCN ≥ or < 2.12 respectively). Accordingly Median TTP was 6.4 months in patients showing increased CISH EGFR GCN whereas it was 3.1 months in those with low CISH EGFR GCN (p = 0.01). Combined results for EGFR FISH and CISH GCN suggested that either FISH or CISH may be used for determination of EGFR GCN substantially depending on each laboratory expertise.

Multiple predictive factors to anti-EGFR monoclonal antibodies treatment have been analyzed since this new class of anti-cancer agents have been introduced in the clinical practice and after the role of K-RAS was clarified in this setting. Among the others a particular role may be attributed to molecular factors that are key regulators of EGFR downstream pathways not influenceable by EGFR upstream directed therapeutic agents (i.e. cetuximab and panitumumab). Tumor NF-kB and B-RAF mutation status are interesting examples of such molecular factors [12,21]. However none of these factors seemed strong enough to translate into clinical practice, mostly because these response determinants singularly taken do not account of all responding (or resistant) patients. The present lack of prospective studies is another evident obstacle to their full introduction into practice. We believe that only a prospective analysis comprehensive of those biological markers suspected to be implicated in response/resistance to EGFR directed therapeutic agents could give researchers and clinicians a full picture of the mechanisms underlying the efficacy of such agents.

## Conclusion

Globally our findings indicate that EGFR GCN as determined either by FISH or CISH may represent a valuable asset in the common effort of better defining, among the group of metastatic K-RAS colorectal cancer patients treated with anti-EGFR antibodies, those more likely to benefit from such a treatment strategy. This approach if further explored seem to possess the required potential to improve the therapeutic effect of this class of molecules in potentially responding patients avoiding unnecessary side effects in resistant cases.

## Competing interests

The authors declare that they have no competing interests.

## Authors' contributions

MS designed the study, performed data analysis and interpretation, wrote the manuscript and final approved it. IB provided study materials, performed and interpreted EGFR FISH/CISH. AM provided study materials, performed and interpreted EGFR FISH/CISH. CP collected patients' data. FL collected patients' data. AZ collected patients' data. FN collected patients' data. AQ collected patients' data. FZ provided study materials. EG analysed and interpreted study data, RB analysed data. TB provided study materials. RL collected patients' data. GM collected patients' data. AF collected patients' data. SC interpreted study findings, supervised the study, approved final version of the manuscript.

All authors read and approved the final manuscript.

## Pre-publication history

The pre-publication history for this paper can be accessed here:

http://www.biomedcentral.com/1471-2407/9/303/prepub
